# Book Review: Directed Motivational Currents and Language Education: Exploring Implications for Pedagogy

**DOI:** 10.3389/fpsyg.2020.616557

**Published:** 2020-11-26

**Authors:** Lixiang Gao, Xu Yang, Honggang Liu

**Affiliations:** ^1^Faculty of Education, Northeast Normal University, Changchun, China; ^2^School of Foreign Languages, Northeast Normal University, Changchun, China

**Keywords:** directed motivational currents, review, quantitative research method, group DMC, intervention study

Directed motivational current (DMC) refers to “an intense motivational drive—or surge—which is capable of stimulating and supporting long-term behavior, such as the learning of an L2” (Dörnyei et al., 2016, p. 2). In the past 8 years, it has been increasingly researched in the field of applied linguistics (Ibrahim and Al-Hoorie, 2019). However, there is still a broad scope to explore DMC in terms of the research methodology, focus, and its applicability in language education. To address these issues, Christine Muir presents the current book titled *Directed Motivational Currents and Language Education: Exploring Implications for Pedagogy*, which moves the research on DMC forward both theoretically and practically.

The book is divided into three sections comprising the following: introducing the basic concepts of DMC, researching DMC via the survey study, and triggering students' DMC in language education. Learners with DMC experience three stages of launching, maintaining, and closing DMC. After demystifying the theoretical issues of DMC, Muir exemplifies how to utilize the questionnaire-based quantitative method to identify language learners' DMC in a worldwide context. Her DMC disposition questionnaire offers the researchers a powerful instrument to confirm language learners' DMC. However, such confirmation of DMC is not the final goal of this book, for Muir also devotes more a considerable part of the book on how to employ the intervention study to trigger students' DMC, especially their group DMC in language education. She demonstrates how the intervention succeeds with reference to the rubrics in the “All Eyes on the Final Product” framework, which evaluates the group DMC (Dörnyei et al., 2016).

Muir's research is prodigious, and there are many things to admire about the book; however, the three major reasons that this book is worth reading are the following. Firstly, this book exemplifies the utilization of the quantitative research method on DMC, which breaks the domination of qualitative methods in this field in the past. Almost all past qualitative studies employed retrospective interviews for data collection because they have the advantage of exploring under-researched areas in social sciences (Dörnyei, 2007). However, these retrospective accounts are disputed quite critically on account of their inability to answer some broad-level questions such as how widely recognizable DMC is. To confirm the learners' DMC in the broader sense can help teachers to design the motivational tasks in the class and to examine the conditions for further triggering students' DMC. Therefore, the quantitative approach becomes the optimal choice to fill this gap. Muir, in this book, introduces the first international questionnaire-based quantitative study, from developing the DMC disposition questionnaire to analyzing the data, which specifies how to do quantitative research on DMC.

Secondly, the book contributes to the exploration of conducting intervention studies to trigger and maintain students' group DMC in classroom contexts—the underexplored and key topic of DMC. Although DMC, in its essence, is highly individualized for the multiple personal goals and learning contexts, it can emerge in the classroom for a group of language learners as a whole (Henry et al., 2015). In classroom teaching, teachers utilize different teaching methods such as project-based instruction and organize various types of group work to motivate students' learning. In this sense, to trigger and sustain students' group DMC is beneficial to enhance their group dynamics (Dörnyei and Murphey, 2003) in the collaborative work and to improve their learning effectiveness, which further helps them to obtain more learning achievements. Thus, the pedagogical implication of DMC that teachers should learn how to make the best use of the conditions that language classrooms provide (e.g., appropriate classroom dynamics and facilitative feedback) via the intervention approach to create the group DMC becomes quite clear. However, few studies in the past have investigated group DMC, especially intervention studies exploring group DMC. To address these gaps, Muir presents the pioneering work, exploring how to purposefully facilitate group DMC with the implementation of an intensive group project based on the “All Eyes on the Final Product” framework (Dörnyei et al., 2016). A series of interventions were performed during the 5-week project, such as defining a clear end goal, providing feedbacks, and linking the project to language studies. In the end, Muir assessed the effectiveness of the whole project based on the criteria of successful intervention in “All Eyes on the Final Product,” finding that each of the criteria put forward by Dörnyei et al. (2016) played a critical role in the success of the intervention.

Thirdly, this book inspires us to explore some possible topics for future DMC research. We can set the questionnaire on DMC experience in different social and cultural learning contexts to validate and extend it. We can also try to replicate the DMC intervention strategies in different contexts of classroom teaching. Another researchable theme is to examine the relationship between DMC and other individual psychological factors (e.g., self-efficacy, self-esteem, etc.).

DMC is a newly proposed concept and is being developed theoretically and practically. The publication of this book serves this developmental goal and makes DMC more popular in the field of applied linguistics or even in the broader arenas of educational psychology and psychology.

## Author Contributions

LG: draft the structure of the review and revisions. XY: drafts and revision. HL: revision and supervision. All authors: contributed to the article and approved the submitted version.

## Conflict of Interest

The authors declare that the research was conducted in the absence of any commercial or financial relationships that could be construed as a potential conflict of interest.
